# The landscape of the A-to-I RNA editome from 462 human genomes

**DOI:** 10.1038/s41598-018-30583-7

**Published:** 2018-08-13

**Authors:** Zhangyi Ouyang, Chao Ren, Feng Liu, Gaole An, Xiaochen Bo, Wenjie Shu

**Affiliations:** 1 Department of Biotechnology, Beijing Institute of Radiation Medicine, Beijing, China; 2 Department of information, The 188th hospital of ChaoZhou, ChaoZhou, China

## Abstract

A-to-I editing, as a post-transcriptional modification process mediated by ADAR, plays a crucial role in many biological processes in metazoans. However, how and to what extent A-to-I editing diversifies and shapes population diversity at the RNA level are largely unknown. Here, we used 462 mRNA-sequencing samples from five populations of the Geuvadis Project and identified 16,518 A-to-I editing sites, with false detection rate of 1.03%. These sites form the landscape of the RNA editome of the human genome. By exploring RNA editing within and between populations, we revealed the geographic restriction of rare editing sites and population-specific patterns of edQTL editing sites. Moreover, we showed that RNA editing can be used to characterize the subtle but substantial diversity between different populations, especially those from different continents. Taken together, our results demonstrated that the nature and structure of populations at the RNA level are illustrated well by RNA editing, which provides insights into the process of how A-to-I editing shapes population diversity at the transcriptomic level. Our work will facilitate the understanding of the landscape of the RNA editome at the population scale and will be helpful for interpreting differences in the distribution and prevalence of disease among individuals and across populations.

## Introduction

A-to-I editing, which is the most common type of RNA editing in metazoans^1^, refers to the process of adenosine (A) deamination to inosine (I), which is then decoded as guanosine (G) in translation. As a post-transcriptional modification mediated by adenosine deaminases acting on RNA (ADARs)^2^, A-to-I editing plays an important role in biological processes by affecting targets such as neuronal receptors^3^, ion transporters^4^, and immune response receptors^5^. A-to-I editing can contribute to transcriptomic and phenotypic diversity by recoding proteins^6^, affecting alternative splicing^7^, modifying microRNAs^8^, and altering microRNA target sites^9^.

The cis-regulation of A-to-I editing has been intensively investigated in *Drosophila*^10–13^, mice^14^ and primates^15,16^. Genetic cis-regulation is important in the comparison of editing levels between genetically diverse outbred mice^14^, different *Drosophila* species^10,12^, and *Drosophila* from a common environment^13^. Recently, researchers identified dozens of differentially edited sites between flies from two opposing slops and showed the relative importance of cis-regulation and environmental regulation in determining these differences^11^. The complex cis- and trans-regulation of RNA editing has been observed by exploring the A-to-I editing profiles of 8,551 human samples from the Genotype-Tissue Expression (GTEx) project^10,15^. Although cis variation in RNA editing has a pronounced impact on RNA secondary structure, it is still largely unknown if there is a difference in the cis- or trans-sequence of A-to-I editing at the human population level.

The 1000 Genomes Project^17^ is characterizing the geographic and functional spectrum of human genetic variation to improve the understanding of genetic contributions to disease. By sequencing 2,504 samples from 26 populations, the consortium recently presented an integrated map of structural variants (SVs). This SV catalogue greatly facilitates studies of population genetics, structural variant demography, functional impacts and disease associations at the DNA level. In contrast to the extensive research on genetic variation in human genomes, how and to what extent A-to-I editing diversifies and shapes population diversity at the RNA level are not fully characterized. In the Geuvadis (Genetic European Variation in Disease) project^18^, proposed by Lappalainen in 2013, mRNA sequencing was performed on 465 lymphoblastoid cell line (LCL) samples from the following populations of the 1000 Genomes Project: CEPH (CEU), Finns (FIN), British (GBR), Toscani (TSI) and Yoruba (YRI). The data from the Geuvadis project allowed us to investigate intra- and inter-population diversity at the transcriptomic level and to explore the landscape of the A-to-I RNA editome at the population scale.

In this study, we identified 16,518 A-to-I editing sites with false detection rate of 1.17% from 462 mRNA-sequencing samples of the Geuvadis project and explored transcriptomic variation among multiple human populations. We compared the probability of RNA editing being shared by two individuals within a population with the probability of sharing by two random individuals from 462 samples. Then, we examined the sharing of editing sites occurring in less than nine individuals across all samples. Our results showed that the sharing of rare RNA editing sites was non-random, suggesting that rare editing sites are likely geographically restricted. Next, we identified 253 highly differentiated editing sites between populations and compared the RNA editing level and the fixation index (*F*_ST_) among five populations. We observed higher diversity of RNA editing between population pairs from different continents relative to those from the same continent, suggesting geographic differentiation of RNA editing. In addition, we analysed the structural motifs of cis- and trans-RNA editing QTLs (edQTLs) in each population separately and discovered the population-specific patterns of these sequence features. Altogether, our results suggested that RNA editing could be used to characterize the nature and structure of populations at the RNA level, which provides insights into how A-to-I editing shapes population diversity at the transcriptomic level. Our study will facilitate the understanding of the RNA editome landscape at the population scale and will be helpful for interpreting differences in the distribution and prevalence of disease among individuals and across populations.

## Results

### Landscape of the A-to-I RNA editome in human populations

To explore the RNA editome and characterize the diversity of RNA editing events in human genomes, we sought to compile a global reference for the RNA editome by utilizing mRNA-seq data from 462 lymphoblastoid cell line (LCL) samples of the Geuvadis project^18^ from the following populations: CEU, FIN, GBR, TSI and YRI (Table S1). These five populations were grouped by the predominant component of ancestry into European (CEU, TSI, GBR and FIN) and African (YRI) populations.

**Figure 1:**
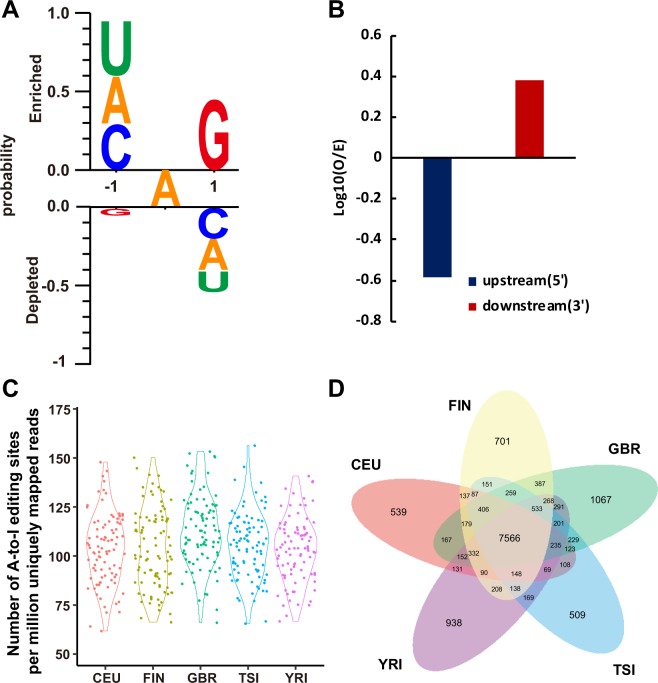
Characterization of RNA editing sites in five populations. (**A**) Sequence preferences for base positions flanking predicted A-to-I editing sites. (**B**) Observed-to-expected (O/E) ratio of the presence of a “G” immediately upstream and downstream of A-to-G editing sites. (**C**) Number of identified RNA editing sites in 462 individuals. Each point represents the number of identified RNA editing sites per million uniquely mapped reads in an individual. We use the number of RNA editing sites per million uniquely mapped reads to avoid the effect of different numbers of mapped reads between individuals. The different colours indicate the different populations. Red for CEU, green for GBR, yellow for FIN, blue for TSI and pink for YRI. We used a violin plot to show the distribution of the number of RNA editing sites in each population. (**D**) Sharing of RNA editing among populations. Different colours indicate the different populations, as in (**A**).

Because A-to-I editing is the most common type of RNA editing in metazoans^1^, we restricted our analysis to A-to-I RNA editing. To identify A-to-I RNA editing candidates among the 462 human genomes from the five populations, we used a pipeline similar to that described in a previous study^19^. To ensure the accuracy of identification, we only retained 16,518 A-to-I editing sites annotated in DARNED and RADAR in the subsequent analyses. Compared with the percentage of non-canonical mismatches identified by our pipeline, the percentage of A-to-I mismatches ranged from 84.86% to 95.25%, with an average of 89.81% (Table S2). The false detection rate for A-to-I mismatches was 1.03% for all sites, ranging from 0.45% to 1.62% for each individual (Table S2). In general, the ratio of the number of G-to-A mismatches to the number of A-to-G mismatches (referred as the noise level) and the magnitude of the ADAR motif (the observed-to-expected (O/E) ratio of the presence of a “G” immediately upstream or downstream of A-to-I editing sites) are used to assess the accuracy of identified RNA editing sites^20^. We assessed the noise level under our identification method, which ranged from 0.06% to 2.25% for each individual, with an average of 0.46% (Table S2). Additionally, we evaluated the occurrence frequency of A, U, C and G bases and the O/E ratio of the presence of a “G” immediately upstream or downstream of A-to-I editing sites. We found that G bases occurred least often at the site −1 bp from A-to-I editing sites and most often at the site 1 bp from those editing sites (Fig. 1A,B); these findings were consistent with previous studies indicating that ADARs show a sequence preference for “G” depletion and “G”enrichment at the 5′ and 3′ nucleotides neighbouring A-to-I editing sites^19,21,22^. Taken together, these results illustrated the accuracy of our procedures in identifying RNA editing sites in human genomes.

In total, we identified 16,518 A-to-I editing sites to generate a reference for the human RNA editome; 15,800 of these sites were located in *Alu* repeats, while 380 were located in repetitive non-*Alu* regions and 338 were located in non-repetitive regions (Table 1). In addition, we annotated A-to-I editing sites using Annovar^23^ with a gene model from GENCODE^24^ (V24) and found that the majority of editing sites were located in 3′ UTRs (47.68%), followed by intronic (26.26%), non-coding RNA (ncRNA, 18.14%) and intergenic regions (4.61%) (Fig. S1). Among the identified RNA editing sites, almost half (8,064; 48.82%) of the sites were common among individuals, while more than one-fifth (3,329; 20.15%) of the sites were low-frequency RNA editing sites, and more than one-third (5,125; 31.03%) of the sites were rare among individuals (Materials and Methods). The ratio of population-specific editing sites and rare editing sites varied by different gene regions. Exonic region holds the highest ratio of population-specific editing sites and rare editing sites, followed by intronic region and intergenic region. UTR5 region also has a higher ratio of population-specific editing sites and rare editing sites than UTR3 region (Fig. S2A,B). Coding region has a significantly higher ratio of population-specific editing sites and rare editing sites than non-coding Alu region (Fig. S2C,D, fisher’s exact test).

**Table 1:** Summary of identified A-to-I editing sites in the autosomes of 462 human genomes.

Samples		CEU	FIN	GBR	TSI	YRI	total
		91	95	94	93	89	462
Total raw bases (Gb)		2080					
Average uniquely mapped reads (±sd)		24,050,000 ± 7,989,095	25,040,000 ± 7,601,546	24,740,000 ± 7,257,506	24,260,000 ± 7,278,070	25,480,000 ± 9,493,014	24,710,000 ± 7,931,331
No. sites overall		10,469	11,590	12,395	10,931	11,469	16,518
Region	UTR3	5,718	5,928	6,119	5,777	5,867	7,875
	intronic	1,997	2,736	3,149	2,344	2,749	4,338
	ncRNA	1,619	1,685	1,829	1,656	1,657	2,436
	intergenic	449	473	523	465	479	761
	downstream	207	210	223	209	228	296
	non-synonymous	22	51	43	36	28	74
	UTR5	43	45	51	43	49	70
	upstream	22	33	32	30	37	46
	synonymous	13	18	21	20	22	31
Location	Alu	10,060	11,097	11,870	10,477	11,004	15,800
	repetitive non-Alu	221	272	273	252	267	380
	non-repetitive	188	221	252	202	198	338
Frequency	rare	920	1,331	1,822	987	1,463	5,125
	low frequency	1,731	2,303	2,567	2,019	2,118	3,329
	common	7,818	7,956	8,006	7,925	7,888	8,064

**Figure 2:**
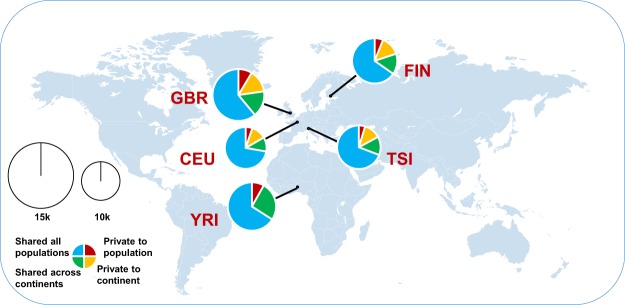
Landscape of the RNA editome in human genomes. Identified RNA editing sites within sampled populations. The area of each pie is proportional to the number of RNA editing sites within a population. The pies are divided into four slices, representing the fraction of RNA editing sites private to a population (red), private to a continental area (orange), shared across continental areas (green), and shared across all populations (blue).

For each population, we identified 10,469, 11,590, 12,395, 10,931 and 11,469 A-to-I editing sites in CEU, FIN, GBR, TSI, and YRI, respectively. We use the number of RNA editing sites per million uniquely mapped reads to avoid the effect of different numbers of mapped reads between individuals. The number of A-to-I editing sites was similar in each population but largely varied among individuals, ranging from 62 per million uniquely mapped reads in one CEU individual to 179 per million uniquely mapped reads in one GBR individual (Figs 1C and S3). We investigated the sharing of all identified RNA editing sites among the populations. Among 16,518 identified RNA editing sites, more than one-fifth of the editing sites (3,754; 22.73%) were private to only one population, and nearly half % of editing sites (7,566; 45.80%) were shared among all populations (Fig. 1D). Within each population, an average of 10,620 editing sites were shared. To explore the distribution of shared RNA editing in each population, we classified editing sites in terms of editing site sharing among populations into private-to-population sites, private-to-continent sites, shared-across-continents sites, and shared-between-all-populations sites. Each population except for YRI presented a similar composition of approximately 67% shared-all-populations sites, 14% shared-across-continents sites, 13% private-to-continent sites, and 6% private-to-population sites (Fig. 2). In YRI, the private-to-continents sites are private-to-population sites because YRI was the only population from Africa included in the analysis. YRI exhibited 66% shared-all-populations sites, 26% shared-across-continents sites, and 8% private-to-population sites. Fisher’s exact test demonstrated that the ratios of private-to-population sites between populations are significantly different except for YRI-GBR and CEU-TSI (Table S3). YRI has significantly higher ratios of private-to-population sites than CEU (fisher’s exact test, one-sided *p*-value < 2.2 × 10^−16^), FIN (fisher’s exact test, one-sided *p*-value = 3.58 × 10^−8^) and TSI (fisher’s exact test, one-sided *p*-value < 2.2 × 10^−16^). Together, these data provide a broad representation of the A-to-I RNA editome of human genomes.

### Geographic restriction of rare A-to-I editing sites within populations

**Figure 3:**
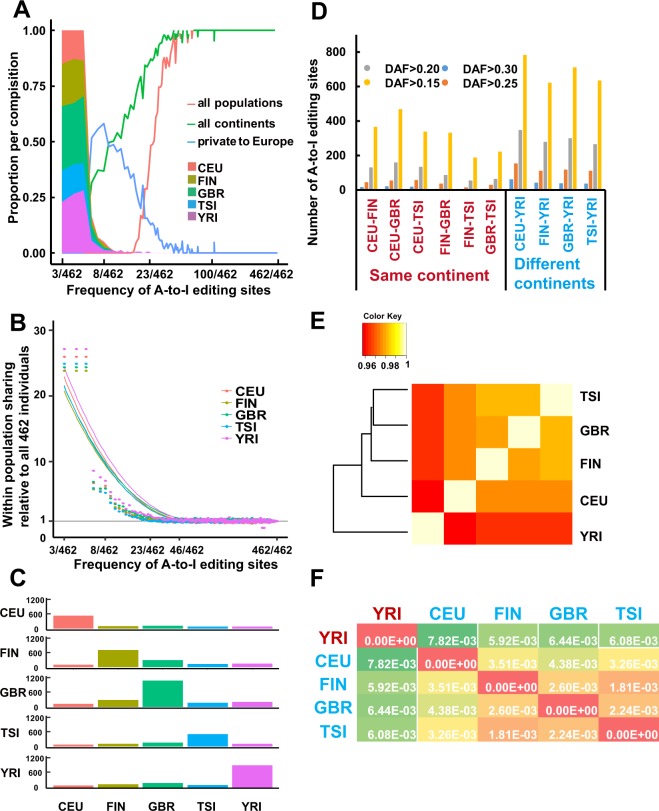
RNA editing sites shared within and between populations. (**A**) Fraction of identified RNA editing sites found in all populations (red line) and on all continents (green line) and those private to Europe (blue line). The stacked area plot shows the cumulative fraction of RNA editing sites private to each population. Red for CEU, green for GBR, yellow for FIN, blue for TSI and pink for YRI. The x-axis is log scaled. (**B**) Excess within-population shared RNA editing as a function of the RNA editing frequency across all samples. The metric is defined as the ratio of the probability of RNA editing sites being shared by two individuals within a population to the probability of RNA editing sites being shared by two random individuals selected from all 462 samples. The x-axis is log scaled. (**C**) Sharing of rarely shared editing sites (found less than nine individuals across the entire sample) between the five populations. Each row represents the distribution across populations for the origin of samples sharing RNA editing sites with the target population (indicated by the left-hand side). (**D**) Number of highly differentiated editing sites with relatively different frequencies between population pairs. We chose 0.15, 0.2, 0.25, and 0.3 as the relative frequencies. (**E**) Hierarchical clustering of RNA editing sites among populations. We used RNA editing sites shared by all populations in the clustering analysis. For each RNA editing site, we chose the average editing level of the samples in the population as the editing level of the population. We defined the distance of editing levels as 1-Spearman’s rho. Then, we plotted the hierarchical clustering using the editing-level distances for all pairs of populations. The plot was generated with the hclust function in R. (**F**) *F*_ST_ between population pairs. We used VCFtools to calculate pairwise *F*_ST_ between populations and chose Weir and Cockerham’s estimator as our estimator.

The reference set of the A-to-I RNA editome provides a prime resource for systematically analysing the patterns in which A-to-I RNA editing sites are shared or monopolized among individuals and populations. To this end, we first investigated the distribution of RNA editing across multiple populations by examining the frequency distribution of RNA editing sites present across all 462 individuals. With an increasing frequency of RNA editing, the proportion of RNA editing shared within a population decreased, and the proportion of RNA editing shared across all populations increased (Fig. 3A). Among the common RNA editing sites, 92% were found in all five populations (7,385 of 8,064), and 73% of the rare RNA editing sites were observed in a single population (3727 of 5,125), suggesting that common RNA editing are prone to be shared across all populations (fisher’s exact test, one-sided *p*-value < 2.2 × 10^−16^) and rare RNA editing sites tend to cluster within private populations (fisher’s exact test, one-sided *p*-value < 2.2 × 10^−16^).

To investigate the pattern of shared RNA editing within populations, we compared the probability of RNA editing sites being shared by two individuals within a population with the probability of RNA editing sites being shared by two random individuals selected from all 462 samples (Fig. 3B). The probability of common RNA editing sites being shared by two individuals within a population was almost equal to the probability of common editing sites being shared by two random individuals from all 462 samples, suggesting that common RNA editing was randomly shared between individuals (Wilcoxon rank sum test, two-sided *p*-value = 0.91). In contrast, the probability of rare editing sites being shared within a population is significantly higher than the probability of rare editing sites being shared by two random individuals across all individual pairs(Wilcoxon rank sum test, one-sided *p*-value = 5.13 × 10^−7^). This result suggested that the sharing of rare editing sites was non-random.

Furthermore, we explored the relationship of the RNA editing sharing between the five populations and the frequency of RNA editing among 462 individuals. We found that population-specific RNA editing (RNA editing private to a population) decreased, while population-shared RNA editing increased as the frequency of RNA editing increased (Fig. S4A). The same result was obtained within each population (Fig. S4B). The ratio of population-specific RNA editing decreased when RNA editing sites occurred in more individuals. Moreover, the population specificity of YRI was slightly higher than the other populations when RNA editing sites occurred in six to eight individuals (Fig. S4B, Wilcoxon rank sum test, one-sided *p*-value = 5.06 × 10^−2^). Next, we examined the distribution of rare RNA editing sites sharing between individuals. We found that GBR exhibited the greatest number of rare editing sites shared within a population, at 1064, followed by YRI, FIN, TSI and CEU. The number of editing sites shared by individuals within a population ranged from 509 to 1,064, whereas the number of rare editing sites shared by individuals from different populations ranged from 81 to 277. Within each population, more than half of the rare RNA editing sites were characterized by population-specific sharing. The sharing of rare editing within population is significantly higher than that sharing between populations (Fig. 3C, Wilcoxon rank sum test, one-sided *p*-value = 3.33 × 10^−4^). These results suggested that the rarely shared editing sites were much more likely to be shared between individuals from the same population. Together, our results revealed that rare editing sites were likely to be geographically restricted.

### High diversity of RNA editing between Europe and Africa

To explore the diversity of RNA editing between continents, we compared the frequency of RNA editing sites between population pairs. We observed some RNA editing sites that were common (present in at least 23 individuals) in RNA editing in one population, but rare (present in less than 9 individuals) in another population; this scenario could be observed between any two populations (Table S4). This result suggested that some RNA editing sites were more likely to occur in one population than in another. To verify this finding, we calculated the difference in the frequency of RNA editing sites between populations and defined highly differentiated editing sites as those with a relatively large difference in frequency of at least 0.3 between population pairs. We obtained 253 highly differentiated RNA editing sites; over two-thirds of these sites (180, 71.15%) were found between YRI and a population from Europe. Hypergeometric hypothesis testing demonstrated that highly differentiated RNA editing sites are significantly enriched in sharing between YRI and European populations (*p*-value = 7.51 × 10^−3^). The number of highly differentiated RNA editing sites in population pairs from different continents was significantly higher than that for population pairs from the same continent (Wilcoxon rank sum test, *p*-value = 6.96 × 10^−3^). We chose 0.15, 0.2 and 0.25 as the relative differences in frequency and obtained consistent results (Fig. 3D). Our results showed that the frequencies of some RNA editing sites in the European population were considerably different from those in the YRI population, suggesting that RNA editing sites described the diversity between continents well.

Next, we wondered whether RNA editing levels could reflect the extent of the differences between different populations. To this end, we compared editing levels between all pairs of the five populations. We calculated the correlation coefficients of editing levels between population pairs using 7,566 shared-all-populations RNA editing sites (Table S5). The editing levels between population pairs from Europe were highly correlated with a mean correlation coefficient of 0.979, ranging from 0.975 (Pearson correlation test, *p*-value < 2.2 × 10^−16^) between the CEU population and FIN population to 0.983 (Pearson correlation test, *p*-value < 2.2 × 10^−16^) between the GBR population and TSI population. However, the correlation coefficient of editing levels between populations from Europe and YRI was slightly lower than that between population pairs from Europe (Fig. 3E). The subtle but substantial difference of editing levels between the European and African populations suggested that the level of editing could faithfully reflect the difference between different continents.

Finally, we performed a pairwise *F*_ST_ analysis of editing sites in the five populations, which can reveal the diversity of RNA editing between populations (Fig. 3F). Typically, a larger *F*_ST_ value indicates greater differentiation between populations. We observed that *F*_ST_ between the populations from Europe and Africa was significantly larger than that between population pairs from Europe (Wilcoxon rank sum test, *p*-value = 4.76 × 10^−3^). *F*_ST_ between population pairs from Europe ranged from 1.81 × 10^−3^, between the FIN and TSI populations, to 4.38 × 10^−3^, between the CEU and GBR populations. However, *F*_ST_ between the YRI population and the various European populations ranged from 5.92 × 10^−3^, between the FIN and YRI populations, to 7.82 × 10^−3^, between the CEU and YSI populations. These results suggested that editing sites could be used to characterize the extent of diversity between populations from different continents well.

### Structural motifs of edQTL editing in human populations

**Figure 4:**
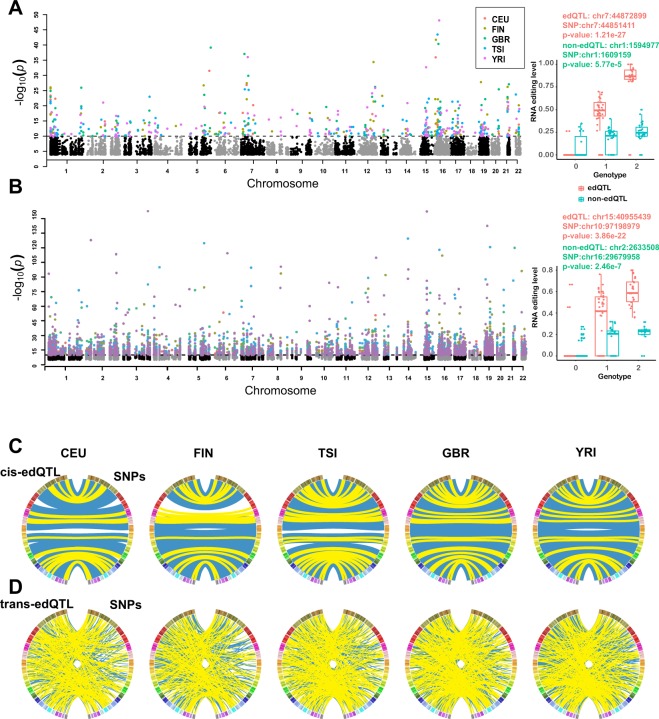
Cis- and trans-edQTLs in populations. (**A**) FDR for each A-to-I editing site in cis-edQTL mapping (left). Grey and black points indicate non-edQTL editing sites. Red for edQTLs in CEU, green for edQTLs in GBR, yellow for edQTLs in FIN, blue for edQTLs in TSI and pink for edQTLs in YRI. An example of cis-edQTL editing sites in CEU (right). The boxplot shows the association of editing levels with the genotype of the associated SNPs. (**B**) FDR for each A-to-I editing site in trans-edQTL mapping (left). Grey and black points represent non-edQTL editing sites. Red for edQTLs in CEU, green for edQTLs in GBR, yellow for edQTLs in FIN, blue for edQTLs in TSI and pink for edQTLs in YRI. An example of trans-edQTL editing sites in CEU (right). The boxplot shows the association of editing levels with the genotype of the associated SNPs. (**C**) Circos plots showing interactions between cis-edQTLs and SNPs. The color bar represents the human chromosomes from chr1 to chr22. The left semicircle represents the location of the cis-edQTL, and the right semicircle represents the location of the SNP associated with cis-edQTL. The population-specific edQTLs are linked by blue lines, and the shared edQTLs are linked by yellow lines. (**D**) Circos plots showing interactions between trans-edQTLs and SNPs. The color bar represents the human chromosomes from chr1 to chr22. The left semicircle represents the location of the trans-edQTL, and the right semicircle represents the location of the SNP associated with trans-edQTL. The population-specific edQTLs are linked by blue lines, and the shared edQTLs are linked by yellow lines.

To explore the association between genomic mutations and the transcriptomic plasticity of RNA editing across populations, we performed edQTL analysis to identify genetic single-nucleotide polymorphisms (SNPs) associated with changes in editing levels. In this analysis, we employed only 445 human lymphoblastoid cell lines for which both genomic and transcriptomic data were available. To address the question of whether there is any difference in edQTLs between populations, we ran MatrixEQTL^25^ separately for each population (Materials and Methods). Limiting the analysis to SNPs within 200 kb upstream and downstream of the RNA editing sites, we identified 85, 90, 128, 128 and 140 cis-edQTLs at a significance threshold of 1e-8 in CEU, FIN, GBR, TSI, and YRI, respectively (Fig. 4A, Table S6). With a significance threshold of 1e-10, we identified 463, 482, 689, 547 and 854 trans-edQTLs in CEU, FIN, GBR, TSI, and YRI, respectively (Figs 4B and S5, Table S7). To verify the edQTLs, we compared the changes in editing levels at edQTL editing sites to those at non-edQTL editing sites, along with the genotypes of the associated SNPs. We found that the editing levels of edQTL editing sites presented a stronger association with the genotypes of the associated SNPs than those of non-edQTLs. For example, for the chr7:44872899 edQTL in the CEU population, the T allele at chr7:44851411 (rs13238404) was associated with a high level of RNA editing, while the A allele nearly abolished RNA editing (Fig. 4A). However, for the chr1:1594977 non-edQTL in the CEU population, there was no marked difference in editing levels associated with the GG, GA, and AA genotypes at chr1:1609159 (rs146575757). The same result was observed for trans-edQTLs (Fig. 4B).

Next, we examined the location of edQTL editing sites in gene regions. The location of edQTL editing sites was similar to that of ordinary A-to-I editing sites. The majority of edQTL editing sites were located in 3′ UTRs, followed by intronic, non-coding RNA and intergenic regions (Fig. S6). In addition, we examined the distribution of edQTL editing sites and associated SNPs. We found that YRI harboured the greatest number of cis-edQTLs and trans-edQTLs. Additionally, YRI exhibited more population-specific edQTL editing sites and a denser association pattern between edQTL editing sites and SNPs than the other populations (Fig. 4C,D).

**Figure 5:**
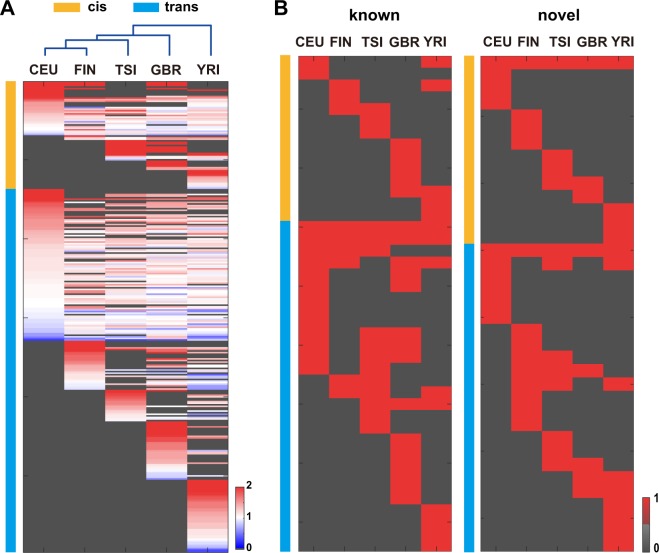
Structural motifs of edQTL editing sites between populations. (**A**) Heatmaps showing the known motifs of edQTL editing sites in populations. Red indicates enrichment relative to background; blue indicates misses relative to the background; and a darker colour indicates a higher degree of enrichment or misses. White indicates that the degree of enrichment is the same as the background; dark grey indicates absence of the motif. Hierarchical clustering using the Spearman distance of the enrichment scores of motifs for all pairs of populations. (**B**) Heatmaps showing the de novo motifs of edQTL editing sites in populations. Red indicates presence of a motif; dark grey indicates the absence of a motif. The left heatmap represents motifs that were associated with a known transcription factor from TRANSFAC. The right heatmap represents novel motifs that were not associated with a known transcription factor from TRANSFAC.

To investigate common sequence and structural features around edQTL editing sites, FIMO^26^ was used to scan motifs in the sequences 250 bp upstream and downstream of edQTL editing sites. For background control, we used the sequences of the 250 bp flanking regions of 16,518 A-to-I editing sites to exclude the structural features of ordinary A-to-I editing sites (Materials and Methods). In total, we identified 69 motifs at cis-edQTL editing sites (cis-motif) and 229 motifs at trans-edQTL editing sites (trans-motif) (Fig. 5A, Table S8). Among the cis-edQTLs, we observed that 34.78% (24 of 69) of the motifs were enriched in a population-specific manner, whereas 27.54% (19 of 69) of the motifs were shared in all five populations. Among trans-edQTLs, we observed that 44.54% (102 of 229) of the motifs were enriched in a population-specific manner, whereas 23.58% (57 of 229) of the motifs are shared in all five populations. Motifs shared among all five populations, such as Pitx2, exhibited similar enrichment across all populations, suggesting that these motifs may be conserved. The higher proportion of population-specific motifs relative to shared-all motifs suggests that the edQTL editing sites of different populations contain different regulatory sequences. Additionally, we performed hierarchical clustering of the enrichment of motifs and found that the enrichment of motifs in YRI was different from that in the other populations (Fig. 5A).

To identify novel transcription factors, the sequences of 500 bp windows centred on the edQTL editing sites were used to identify sequence motifs with MEME^27^ and DREME^28^ (Materials and Methods). The sequences of the 250 bp flanking regions of all 16,518 A-to-I editing sites were used to generate a second-order Markov model as a background control. We identified 28 novel cis-motifs and 51 novel trans-motifs with a very stringent cut-off (P < 1e-8). We annotated the most likely transcription factor for each motif by comparing it with public transcription factor database TRANSFAC^29^ using TOMTOM^30^ (Table S9). A total of 53.57% of the de novo cis-motifs (15 of 28) and 54.90% of the de novo trans-motifs (28 of 51) were associated with a known transcription factor. Within these motifs associated with a known transcription factor, 85.71% of cis-motifs (12 of 14) showed a population-specific pattern, whereas only 53.57% of trans-motifs (15 of 28) showed a population-specific pattern (Fig. 5B). Within de novo motifs, 85.71% of cis-motifs (12 of 14) showed a population-specific pattern, whereas only 65.21% of trans-motifs (15 of 23) showed a population-specific pattern (Fig. 5B). The de novo motifs presented a similar population-specific pattern to the known motifs.

Since most A-to-I editing sites are located in Alu regions, we performed the same FIMO and MEME analyses using the Alu sequences as background control to exclude the structural features of Alu regions (Tables S10, S11, Materials and Methods). The population-specific pattern was still observed (Fig. S7). Taken together, the results indicated the population-specific patterns of cis- and trans-sequences of A-to-I editing.

## Discussion and Conclusions

Plentiful genome-wide SNP data have made the systematic study of population genetics viable and feasible. However, the study of population genetics is not well understood at the RNA level based on transcriptome data. To explore population diversity at the transcriptomic level, we used 462 mRNA-sequencing samples from five populations of the Geuvadis project^18^ and identified a total of 16,518 A-to-I editing sites with false detection rate of 1.03%. Examination of the sharing of the identified RNA editing sites within each population and between populations revealed that the landscape of the human RNA editome could reflect the unique nature of human populations. Thus, we present a broad representation of the A-to-I RNA editome of human genomes, which will be of immense use to future studies investigating RNA editing at the population level.

We evaluated the pattern of shared RNA editing among individuals and populations and explored the geographic diversity of rare RNA variants. Exploration of the frequency distribution of RNA editing sites across all 462 individuals revealed that rare RNA editing sites tended to cluster within private populations. Furthermore, we examined the distribution of rare editing sites across all samples and found that rare editing sites were much more likely to be shared between individuals from the same population. Our results suggested that rare editing sites were likely geographically restricted and non-randomly shared within populations.

Then, we explored the diversity of RNA editing between populations from different continents. We identified 253 highly differentiated editing sites with a relatively large difference in frequency of at least 0.3 between population pairs. The majority of highly differentiated editing sites between populations were shared by population pairs from different continents. Furthermore, we performed an analysis of differences in editing levels and an *F*_ST_ analysis between population pairs. Our analyses revealed high concordance between population pairs from the same continent in terms of both editing levels and *F*_ST_ and showed slight but substantial differences between population pairs from different continents for both parameters. The results demonstrated that the fascinating characteristics of RNA editing could illustrate the diversity between populations from different continents.

Finally, we explored the association between genetic SNPs and transcriptomic A-to-I editing sites in human populations via cis- and trans-edQTLs. We observed that YRI presented more cis- and trans-edQTL editing sites than the other populations, especially for population-specific edQTL editing sites. Furthermore, the structural motifs of edQTL editing sites were enriched in a population-specific manner, suggesting the existence of different regulatory patterns between populations. Two other recent studies also performed RNA editing analysis on the same RNA-seq data from these human populations that we used. Xiao’s group discovered differences in RNA editing prevalence within populations and low-level differences in the shared editing sites of the populations^31^. They demonstrated that the strikingly different gene expression of ADARs between populations does not account for these editing differences within populations but that AGO2-miRNA targeting could affect mRNA abundance and in turn alter the observed editing levels. Xing’s group combined edQTL analysis with allele-specific RNA editing (ASED) analysis in human populations. They discovered that SNPs associated with the variation in RNA editing function more closely to their respective editing sites and that some of these SNPs are linked to genome-wide association study (GWAS) signals of complex traits or diseases among genetically distinct individuals^32^. Their work demonstrated the important functional impact of RNA editing on biology and disease in human cells. The population-specific pattern of edQTL editing sites could aid in interpreting the distribution of disease among populations. These results suggest that genetic mutation or RBPs other than ADARs affect the differences in RNA editing between populations, but how and to what extent this regulation modulates RNA editing have yet to be explored.

The 1000 Genomes Project Consortium^17^ identified millions of genetic variants in humans and interpreted their functional effects to understand the genetic basis of variation in human traits. In addition, the Consortium showed that the majority of rare variants was shared by individuals from the same population and that rare variants had arisen more recently than distinct populations. These results demonstrated that rare (<0.5%) variants are highly informative regarding population structure and recent demography, suggesting that these variations can reflect substantial local differentiation, in line with population history. Genetic variability is considered key to evolution. Strikingly, our analysis demonstrated that rare RNA editing sites can illustrate the properties of populations well, consistent with rare genetic variants^17,33^. The only difference is that rare genetic variants characterize the nature of a population at the DNA level, whereas rare transcriptomic RNA editing characterizes the nature of a population at the RNA level. Consistent results were obtained regardless of the application of relatively loose filters, requiring RNA editing sites with coverage of at least five reads and at least two edited reads or the selection of the strict GIREMI^34^ method to identify RNA editing sites, which demonstrated that our results were reliable and were not affected by the adopted filtering steps or identification methods (Figs S8–S10). In fact, RNA editing can rapidly respond to environmental stress before any genetic changes^35–37^; moreover, the differences in editing levels between parents are largely maintained in F1 hybrid alleles, indicating the role of RNA editing in evolution^12^. Compared with genetic variation, the level of RNA editing may range from almost zero to 100%, which makes RNA editing more flexible during evolution. The variable RNA editing level increases phenotypic plasticity and provides an evolutionary advantage for long-term acclimatization.

Taken together, our findings provide insights into the process of how A-to-I editing shapes population diversity at the transcriptomic level. Our characterization of RNA editing within and between populations suggests that A-to-I editing sites can depict the nature and structure of a population at the RNA level well, which provides a transcriptomic perspective for the exploration of population genetics. Our work will facilitate the understanding of the RNA editome at the population level and will be helpful for interpreting differences in the distribution and prevalence of disease among individuals and across populations.

## Materials and Methods

### Identification of A-to-I editing sites in human genomes

The Geuvadis project^18^ sequenced 465 lymphoblastoid cell line (LCL) RNA-seq samples and included 462 individuals with available mRNA after the application of quality control measures. We obtained mRNA-seq data from the 462 Geuvadis project samples, which came from the following populations of the 1000 Genomes Project^17^: CEU, FIN, GBR, TSI and YRI (Table S1). These five populations, which included 89–95 samples per population, were grouped by the predominant component of ancestry into European (CEU, TSI, GBR and FIN) and African (YRI) populations. To identify A-to-I editing sites, we applied separate samples method that described in a previous study^19^. The only modification of this methodology was the choice of STAR as mapping software instead of BWA, because STAR presents a high mapping speed and produces accurate alignment of contiguous and spliced reads. Additionally, STAR and GATK are the best practices for calling variants in RNA-seq data. The other variant filters were the same as in the previous study^19^. First, we used STAR (version 2.5.2b)^38^ to align RNA-seq reads to the hg19 human reference with default parameters. Then, we only considered uniquely mapped reads with tag ‘NH:i:1’ and used Picard (https://broadinstitute.github.io/picard/) to remove duplicated reads that mapped to the same location. Reads with a mapping quality <20 were removed by SAMtools (Version: 1.3.1) with the parameter ‘−q 20’. Next, we called variants using the GATK (version 3.5.0)^39^ HaplotypeCaller tool with the options stand_call_conf of 20 and stand_emit_conf of 0. Then, we removed all variants present in dbSNP (except for SNPs of the molecular type ‘cDNA’; database version 137, http://www.ncbi.nlm.nih.gov/SNP/) and the 1000 Genomes Project database. We discarded variants located within the first 6 bp from either end of a sequence read. In non-*Alu* regions, we removed sites within simple repeats according to RepeatMasker annotation, discarded intronic candidates if they were located within 4 bp of all known splicing junctions according to Ensembl gene annotations, and removed sites in homopolymer runs of ≥5 bp. We also excluded variant sites in regions showing high similarity to other parts of the genome using the BLAST-like alignment tool (BLAT)^40^. Finally, we required each RNA editing site to exhibit coverage of at least ten reads and at least three edited reads and to be present in more than three individuals of a population. We inferred the editing type of each site based on the strand of overlapping annotated genes and retained only A-to-I editing sites annotated in the DARNED and RADAR databases for subsequent analysis.

### Validation and annotation of RNA editing sites

To validate the identified A-to-I editing events, we compared our editing sites with the RNA editing databases DARNED^41^ and RADAR^42^, which include 333,214 and 259,5361 events, respectively. In total, the two databases compile 2,598,505 editing sites. Among our identified A-to-I editing sites, 11,511 (68.46%) and 16,499 (98.12%) were found in the DARNED and RADAR databases, respectively. Collectively, 16,518 (98.23%) of our identified editing sites were present in these two databases.

To assess the error rate arising from our identification strategy, we calculated the false detection rate of this methodology. Assuming that all non-A-to-I mismatches were false and that the error rate for all 12 mismatch types was equal, we use this formula to estimate our false detection rate:

$$\frac{(1-AG \% )/11}{AG \% }=\frac{nonAG \% }{AG \% }=\frac{nonAG/All}{AG/All}=\frac{nonAG}{AG}$$

This formula denotes the ratio of the number of any other one type of non-A-to-G mismatches to that number of A-to-G mismatches. Where AG% is the ratio of A-to-G editing sites in all identified RNA editing sites, nonAG% is the ratio of any other one type of non-A-to-G editing sites in all identified RNA editing sites (referred to as nonAG%), All is the number of identified RNA editing sites, AG is the number of A-to-G editing sites, nonAG is the number of any other one type of non-A-to-G editing sites.

For each RNA editing site, functional annotation was performed using Annovar^23^, with the gene definitions of GENCODE^24^ (V24) (including exonic, intronic, 5′UTR, 3′UTR, and intergenic). The A-to-I editing sites within exonic regions were further defined as “synonymous” or “non-synonymous” based on whether they change amino acids in the protein products.

### Classification of RNA editing

We defined the frequency of an RNA editing site by calculating how many times that RNA editing site occurred across all 462 samples. In terms of the frequency of RNA editing, we classified RNA editing sites into three categories: rare editing sites (present in less than 9 individuals), low-frequency editing sites (present in between 9 individuals and 22 individuals) and common editing sites (present in more than 22 individuals). In terms of the sharing number of population, RNA editing sites can be classified into population-specific, share-in-two-populations, share-in-three-populations, share-in-four-populations and share-in-all-populations.

### Geographical differentiation of rare RNA editing sites

To further verify whether A-to-I editing sites could reflect a population’s characteristics, we measured the ratio of the probability of editing sites being shared by two individuals within a population compared with the probability of being shared by two random individuals selected from all 462 samples.

### Diversity of RNA editing between continents

We employed RNA editing sites shared by all populations to analyse the divergence of RNA editing levels between populations. For each RNA editing site, we chose the average editing level of individuals in that population as the editing level of the population. We defined the distance of editing levels as 1-Spearman’s rho between the editing levels of two populations. Then, we performed hierarchical clustering analysis of editing levels for all population pairs using the function hclust in R.

To compare the level of differentiation between populations, we calculated the fixation index (*F*_ST_), a measure of population differentiation due to genetic structure. We used VCFtools^43^ to estimate *F*_ST_ for each pairwise population comparison and chose Weir and Cockerham’s weighted method as our estimator.

### Identification of highly differentiated editing sites between populations

We defined highly differentiated editing sites as those with a relatively large difference in frequency of at least 0.3 between population pairs. We discovered the number of these editing sites shared by each population pair. In total, we identified 253 highly differentiated editing sites across the five populations. We also chose 0.15, 0.2 and 0.25 as relative frequencies and obtained 4,666, 1,824, and 736 highly differentiated editing sites.

### Identification of edQTLs

For genome-wide mapping of edQTLs in each population, the following criteria were applied: (1) For RNA editing sites in each population, we removed sites with low variance (coefficient of variance <0.8). (2) In each population, only variants that were heterogeneous in at least one sample in the population were used to map edQTLs. Variants located within a 400 kb window centred at the editing site were used to map cis-edQTLs. (3) MatrixEQTL was employed to map cis- and trans-edQTLs. The results were filtered separately with significance thresholds of 1e-8 and 1e-10. FDRs were calculated with MatrixEQTL. (4) For each RNA editing site, the SNP with smallest p value located at the shortest distance was chosen as the edQTL SNP. For trans-edQTLs with the same smallest p-value, the edQTL SNP was chosen randomly among these edQTLs. The associations between edQTL editing sites and SNPs were drawn with Circos^44^.

### Structural motifs of edQTL editing sites

For each edQTL editing site, the sequence of the 500 bp window centred on the editing site was used to scan potential transcript factor binding sites. FIMO was employed to scan motifs from TRANSFAC in the window against three different backgrounds (the backgrounds provided by FIMO and TRANSFAC and the background generated from the window sequence). The p-values for each motif occurrence are converted to q-values following the method of Benjamini and Hochberg. Binding sites that passed the q-value < 1e-6 were counted as potential TF binding sites. The density of a transcription factor was defined as the

$$\frac{Number\,of\,potential\,TFBSs\ast Length\,of\,motif}{Total\,length\,of\,window\,sequence}\ast 100000.$$

To illustrate the relative enrichment of TF binding sites in the five population, we used the 500 bp window sequences centred on all 16,518 RNA editing sites as a control. The enrichment of TFs in each population was defined as

$${\mathrm{log}}_{2}(\frac{Density\,in\,population}{Density\,in\,control}+1).$$

The populations were clustered using Spearman correlation of TF enrichment as a distance metric. For the Alu region background control, we randomly selected the 500 bp window sequence centred on Alu regions.

For each edQTL editing site, the 500 bp window centred on the editing site was used to identify the sequence motif with MEME and DREME. For the background control, we employed two second-order Markov models generated from the 500 bp window sequences centred on the 16,518 RNA editing sites and Alu regions separately. The width of the motifs identified by MEME was limited to 10 bp. All motifs identified with an E-value < 1e-8 were compared with the motifs in TRANSFAC using TOMTOM.

## Electronic supplementary material

Supplementary information

Supplementary tables

## Data Availability

The RNA editing sites identified among the 462 human genomes from the five populations have been deposited with the Gene Expression Omnibus under accession ID GSE103294.
