# Supplementary material for: The landscape of the A-to-I RNA editome from 462 human genomes
Source: Sci Rep. 2018 Aug 13;8:12069. doi: 10.1038/s41598-018-30583-7 (PMC6089959; doi:10.1038/s41598-018-30583-7)
Supplement: Supplementary file 1 — Supplementary information [file 41598_2018_30583_MOESM1_ESM.pdf]

The landscape of the A-to-I RNA editome from 462 human genomes

Zhangyi Ouyang[1], Chao Ren[1], Feng Liu[2], Gaole An[1], Xiaochen Bo[1*], Wenjie Shu[1*]

[1]Department of Biotechnology, Beijing Institute of Radiation Medicine, Beijing, China

[2]Department of information, The 188th hospital of ChaoZhou, ChaoZhou, China

*To whom correspondence should be addressed. Tel.: +86 10 6693 2211; Fax: +86 10 6821 0077; Email: shuwj@bmi.ac.cn

Correspondence may also be addressed to Xiaochen Bo. Email: boxc@bmi.ac.cn

Supporting Information Legends

Supplementary Figure S1. Distribution of the identified RNA editing sites in different genic regions.

Supplementary Figure S2. The sharing of RNA editing in different gene regions.

(A) The ratio of rare, low frequency and common RNA editing sites in different gene regions.

(B) The ratio of rare, low frequency and common RNA editing sites in different gene regions.

(C) The sharing of RNA editing in coding region and non-coding region (fisher's exact test, one-sided p-value).

(D) The ratio of rare, low frequency and common RNA editing sites in coding region and non-coding region (fisher's exact test, one-sided p-value).

Supplementary Figure S3. Number and editing levels of identified RNA editing sites in 462 individuals.

(A) Number of identified RNA editing sites in each individual.

(B) Distribution of the identified RNA editing sites within populations.

(C) RNA editing level of each individual.

Supplementary Figure S4. Relationship between the sharing and frequency of RNA editing.

Supplementary Figure S5. RNA editing level of edQTL editing sites in each population.

Supplementary Figure S6. Distribution of identified edQTL editing sites in different genic regions.

Supplementary Figure S7. Structural motifs of edQTL editing sites between populations using random Alu regions as background control.

(A) Heatmaps showing the known motifs of edQTL editing sites in populations. Red indicates enrichment relative to background; blue indicates misses relative to the background; and a darker colour indicates a higher degree of enrichment or misses. White indicates that the degree of enrichment is the same as the background; dark grey indicates absence of the motif. Hierarchical clustering using the Spearman distance of the enrichment scores of motifs for all pairs of populations.

(B) Heatmaps showing the de novo motifs of edQTL editing sites in populations. Red indicates presence of a motif; dark grey indicates the absence of a motif. The left heatmap represents motifs that were associated with a known transcription factor from TRANSFAC. The right heatmap represents novel motifs that were not associated with a known transcription factor from TRANSFAC.

Supplementary Figure S8. Landscape of the RNA editome in human genomes.

Identified RNA editing sites within the sampled populations. The area of each pie is proportional to the number of RNA editing sites within a population. The pies are divided into four slices, representing the fraction of RNA editing sites private to a population (red), private to a continental area (orange), shared across continental areas (green), and shared across all populations (blue). Dashed lines indicate populations sampled outside of their ancestral continental region. (A) We required each A-to-I editing site with coverage of at least five reads and at least two edited read. (B) We

identified A-to-I editing sites using the GIREMI method.

Supplementary Figure S9. RNA editing sites shared within and between populations. We required each RNA editing site to present coverage of at least five reads and at least two edited read.

(A) The fraction of identified RNA editing sites found in all populations (red line) and on all continents (yellow line) and those private to Europe (blue line). The stacked area plot shows the cumulative fraction of RNA editing sites private to each population. Red for CEU, green for GBR, yellow for FIN, blue for TSI and pink for YRI. The x-axis is log scaled.

(B) Excess within-population shared RNA editing as a function of the RNA editing frequency across all samples. The metric is defined as the ratio of the probability of RNA editing sites shared by two individuals within a population to the probability of RNA editing sites shared by two random individuals selected from all 462 samples. The x-axis is log scaled.

(C) Sharing of rarely shared editing sites (those found less than nine individuals across the entire sample) between the five populations. Each row represents the distribution across populations for the origin of samples sharing RNA editing sites with the target population (indicated by the left-hand side).

(D) Number of highly differentiated editing sites with relatively different frequencies between population pairs. We chose 0.15, 0.2, 0.25, and 0.3 as the relative frequencies.

(E) Hierarchical clustering of RNA editing sites among populations. We used the RNA editing sites shared by all populations in the clustering analysis. For each RNA editing site, we chose the average editing level of the samples in that population as the editing level of the population. We defined the distance of editing levels as 1-Spearman’s rho. Then, we plotted the hierarchical clustering using the editing-level distances for all pairs of populations. The plot was generated with the hclust function in R.

(F) F_{ST} between population pairs. We used VCFtools to calculate pairwise F_{ST} between populations and chose Weir and Cockerham's estimator as our estimator.

Supplementary Figure S10. RNA editing sites shared within and between populations. We identified A-to-I editing sites using the GIREMI method.

(A) Fraction of identified RNA editing sites that were found in all populations (red line) and on all continents (yellow line) and those private to Europe (blue line). The stacked area plot shows the cumulative fraction of RNA editing sites private to each population. Red for CEU, green for GBR, yellow for FIN, blue for TSI and pink for YRI. The x-axis is log scaled.

(B) Excess within-population shared RNA editing as a function of the RNA editing frequency across all samples. The metric is defined as the ratio of the probability of RNA editing sites being shared by two individuals within a population to the probability of RNA editing sites being shared by two random individuals selected from all 462 samples. The x-axis is log scaled.

(C) Sharing of rarely shared editing sites (found less than nine individuals across the entire sample) between the five populations. Each row represents the distribution across populations for the origin of samples sharing RNA editing sites with the target population (indicated by the left-hand side).

(D) Number of highly differentiated editing sites with relatively different frequencies between population pairs. We chose 0.15, 0.2, 0.25, and 0.3 as the relative frequencies.

(E) Hierarchical clustering of RNA editing sites among populations. We used RNA editing sites shared by all populations in the clustering analysis. For each RNA editing site, we chose the average editing level of the samples in this population as the editing level of the population. We defined the distance of editing levels as 1-Spearman's rho. Then, we plotted the hierarchical clustering using the editing-level distances for all pairs of populations. The plot was generated with the hclust function in R.

(F) F_{ST} between population pairs. We used VCFtools to calculate pairwise F_{ST} between populations and chose Weir and Cockerham's estimator as our estimator.

Table S1. Information for 462 individuals from the Geuvadis Project.

Table S2. Mapping information and identification of RNA editing for 462 human genomes.

Table S3. Fisher's exact test of the ratios of private-to-population sites between populations. Two sided p-value is given in the table.

Table S4. Number of RNA editing sites common in one population (population 1) and rare in another population (population 2).

Table S5. Pearson correlation coefficients of mean editing levels between populations.

Table S6. The cis-edQTL editing sites and their associated SNPs in five populations.

Table S7. The trans-edQTL editing sites and their associated SNPs in five populations.

Table S8. The enrichment of structural motif identified by FIMO using all 16,518 RNA editing sites as control. The enrichment of TFs in each population was defined as $\log_2(1+(\text{Density in population}/\text{Density in control}))$.

Table S9. The $-\log_{10}$(E-value) of structural motif identified by MEME using all 16,518 RNA editing sites as control. All motifs identified with E-value < 1e-8 were compared with motifs in TRANSFAC using TOMTOM.

Table S10. The enrichment of structural motif identified by FIMO using random Alu regions as control. The enrichment of TFs in each population was defined as $\log_2(1+(\text{Density in population}/\text{Density in control}))$.

Table S11. The $-\log_{10}$(E-value) of structural motif identified by MEME using random Alu region as control. All motifs identified with E-value < 1e-8 were compared with motifs in TRANSFAC using TOMTOM.

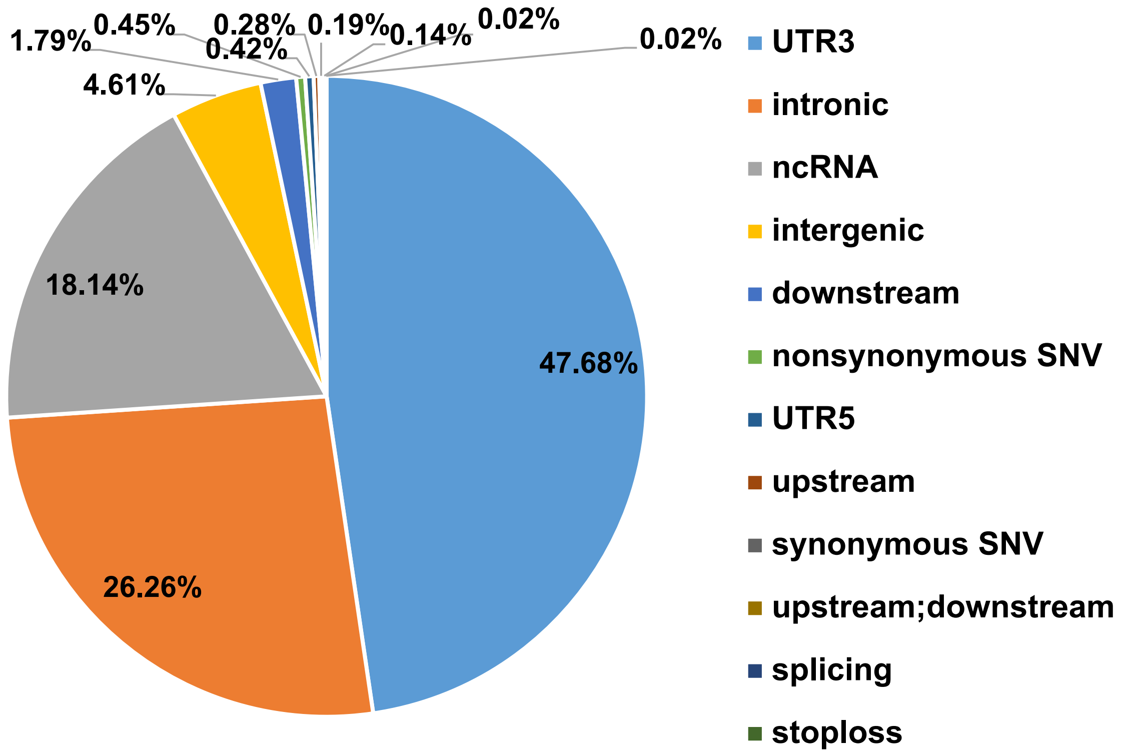
1.79%
0.45%
0.28%
0.42%
0.19%
0.14%
0.02%
0.02%
4.61%
18.14%
47.68%
26.26%
UTR3
intronic
ncRNA
intergenic
downstream
nonsynonymous SNV
UTR5
upstream
synonymous SNV
upstream;downstream
splicing
stoploss

Figure S1

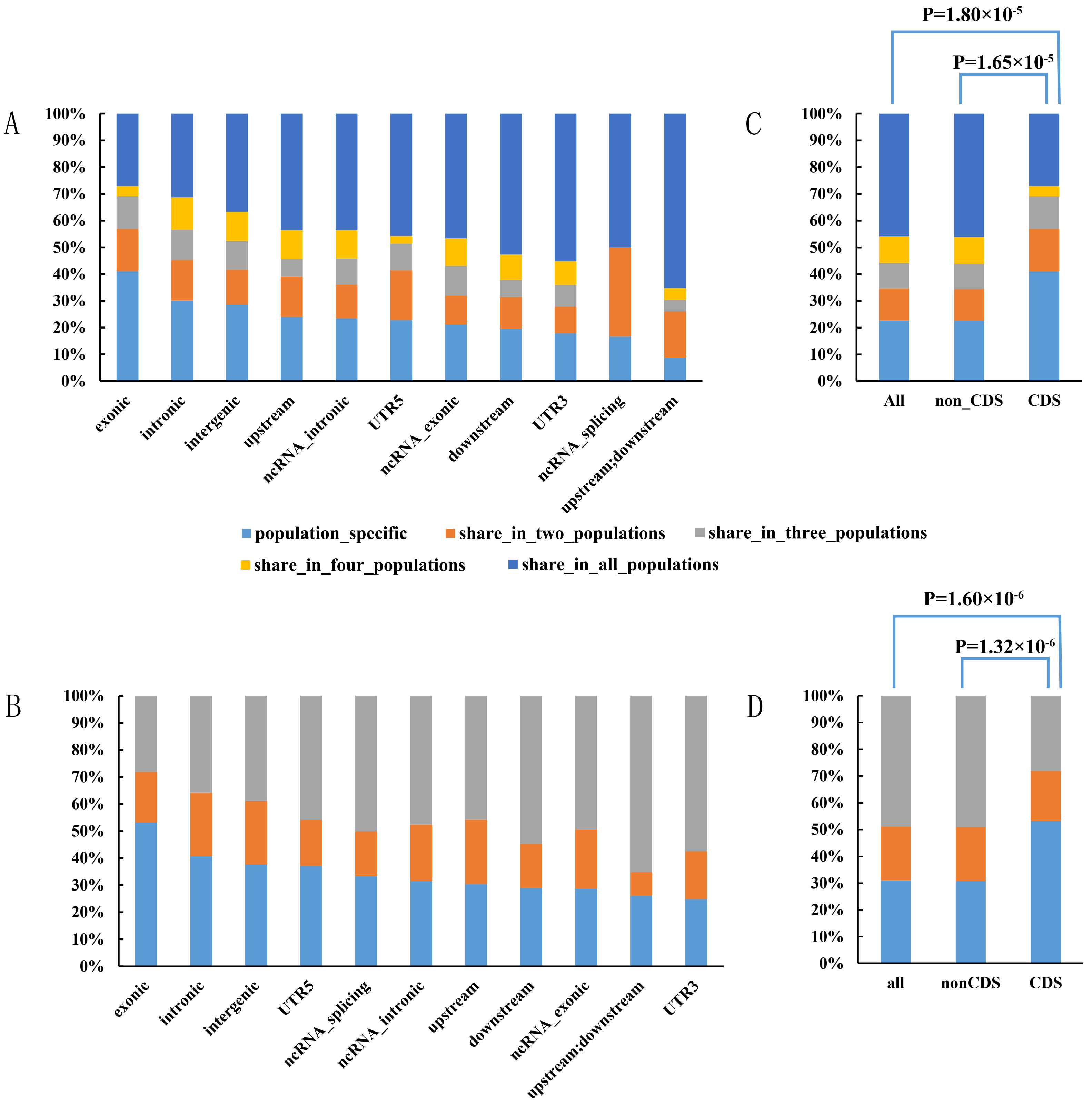
A
B
C
D
P=1.80×10-5
P=1.65×10-5
P=1.60×10-6
P=1.32×10-6
100%
90%
80%
70%
60%
50%
40%
30%
20%
10%
0%
exonic
intronic
intergenic
upstream
ncRNA_intronic
UTR5
ncRNA_exonic
downstream
UTR3
ncRNA_splicing
upstream;downstream
All
non_CDS
CDS
all
nonCDS
population_specific
share_in_two_populations
share_in_three_populations
share_in_four_populations
share_in_all_populations
rare
low frequency
common

Figure S2

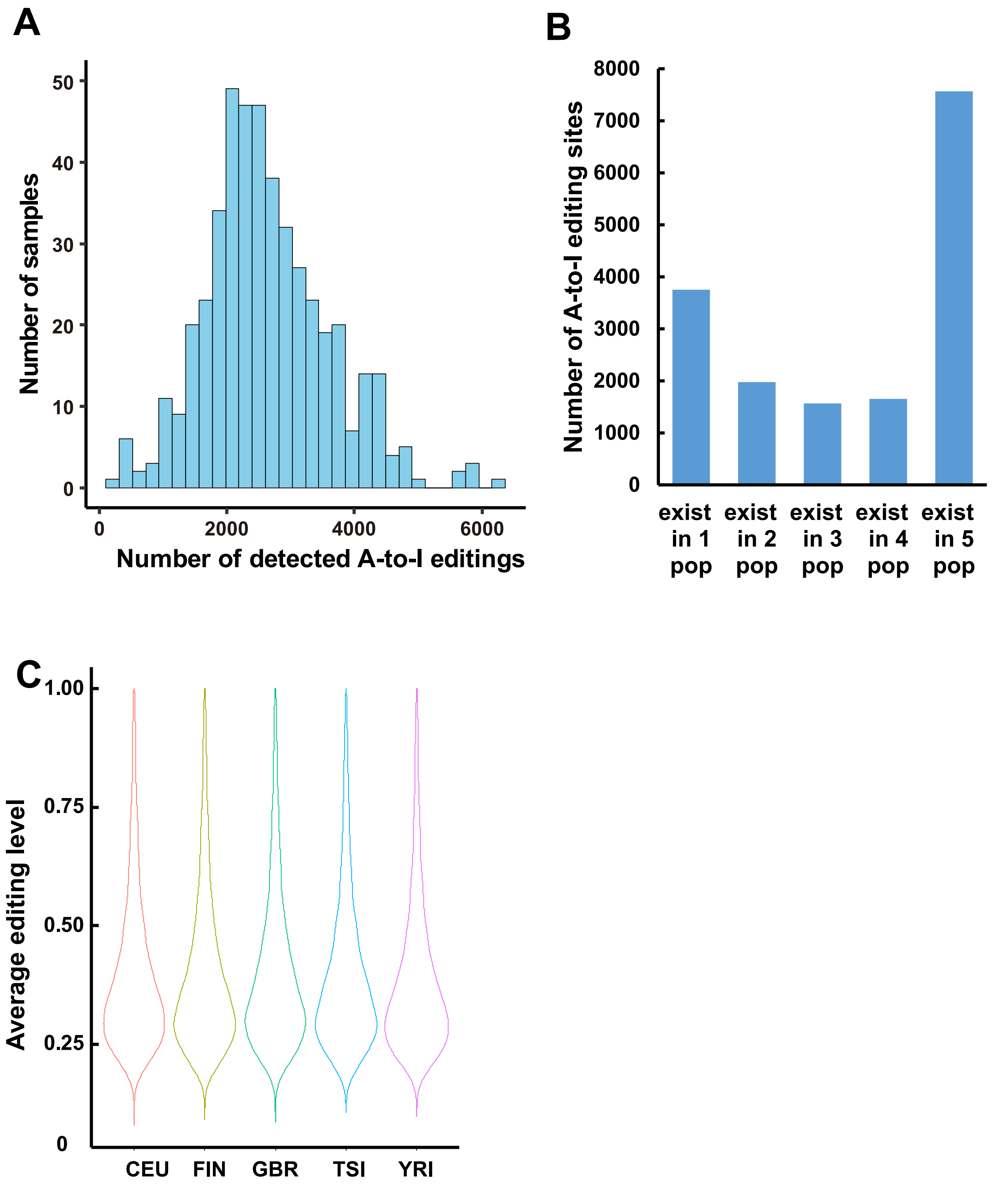
A
Number of samples
0
10
20
30
40
50
0
2000
4000
6000
Number of detected A-to-I editings
B
Number of A-to-I editing sites
0
1000
2000
3000
4000
5000
6000
7000
8000
exist in 1 pop
exist in 2 pop
exist in 3 pop
exist in 4 pop
exist in 5 pop
C
Average editing level
0
0.25
0.50
0.75
1.00
CEU
FIN
GBR
TSI
YRI

Figure S3

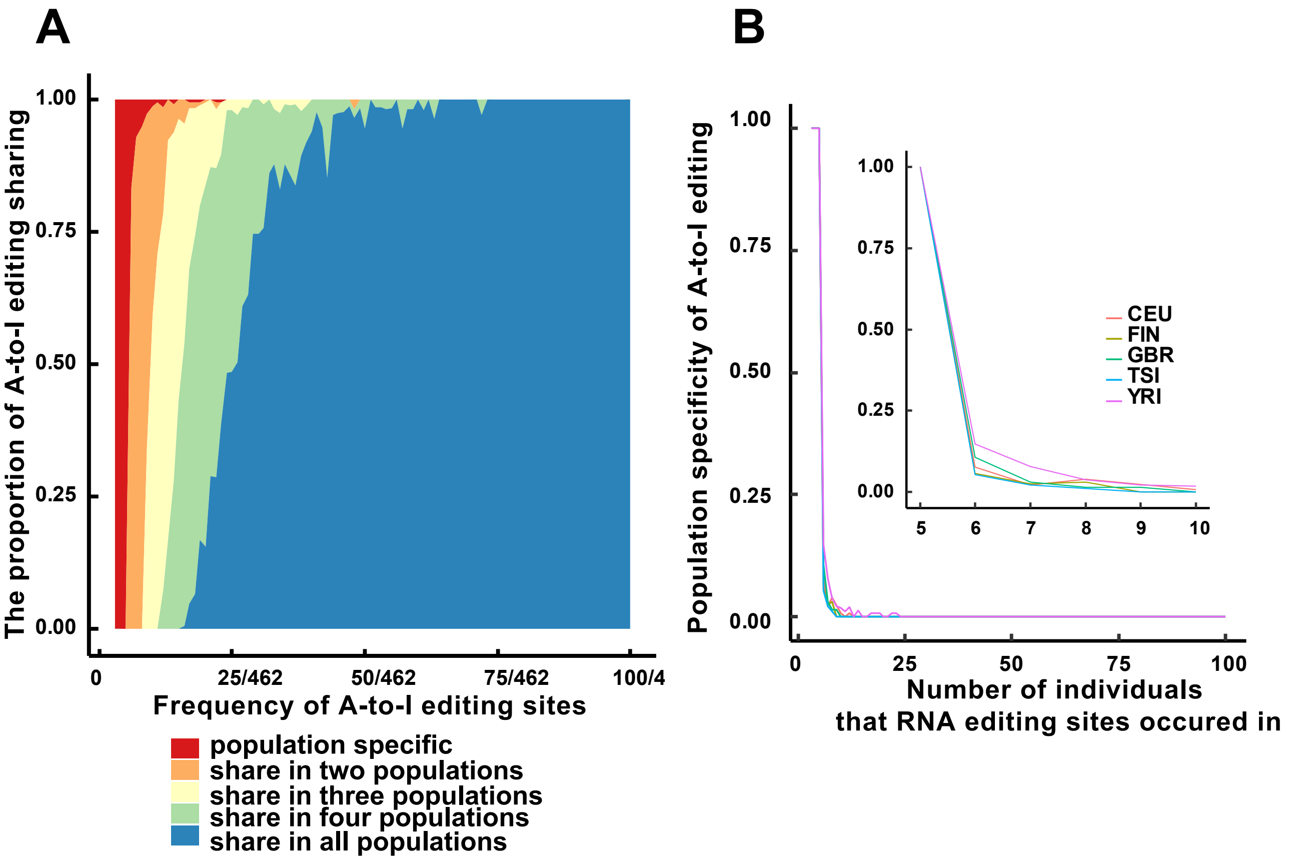

A
1.00
0.75
0.50
0.25
0.00
The proportion of A-to-I editing sharing
0
25/462
50/462
75/462
100/4
Frequency of A-to-I editing sites
population specific
share in two populations
share in three populations
share in four populations
share in all populations
B
Population specificity of A-to-I editing
1.00
0.75
0.50
0.25
0.00
0
25
50
75
100
Number of individuals
that RNA editing sites occured in
1.00
0.75
0.50
0.25
0.00
5
6
7
8
9
10
CEU
FIN
GBR
TSI
YRI

Figure S4

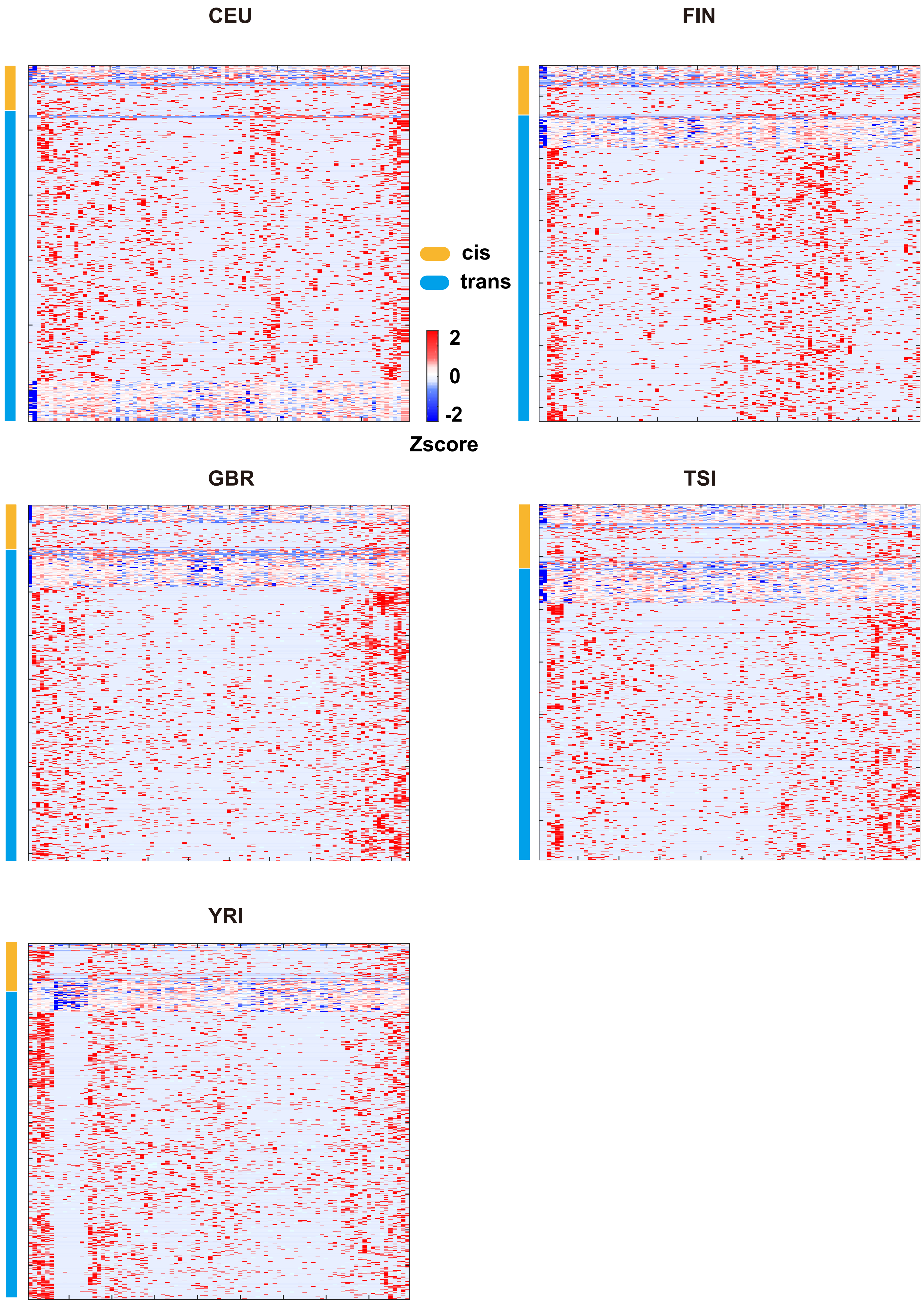

Figure S5

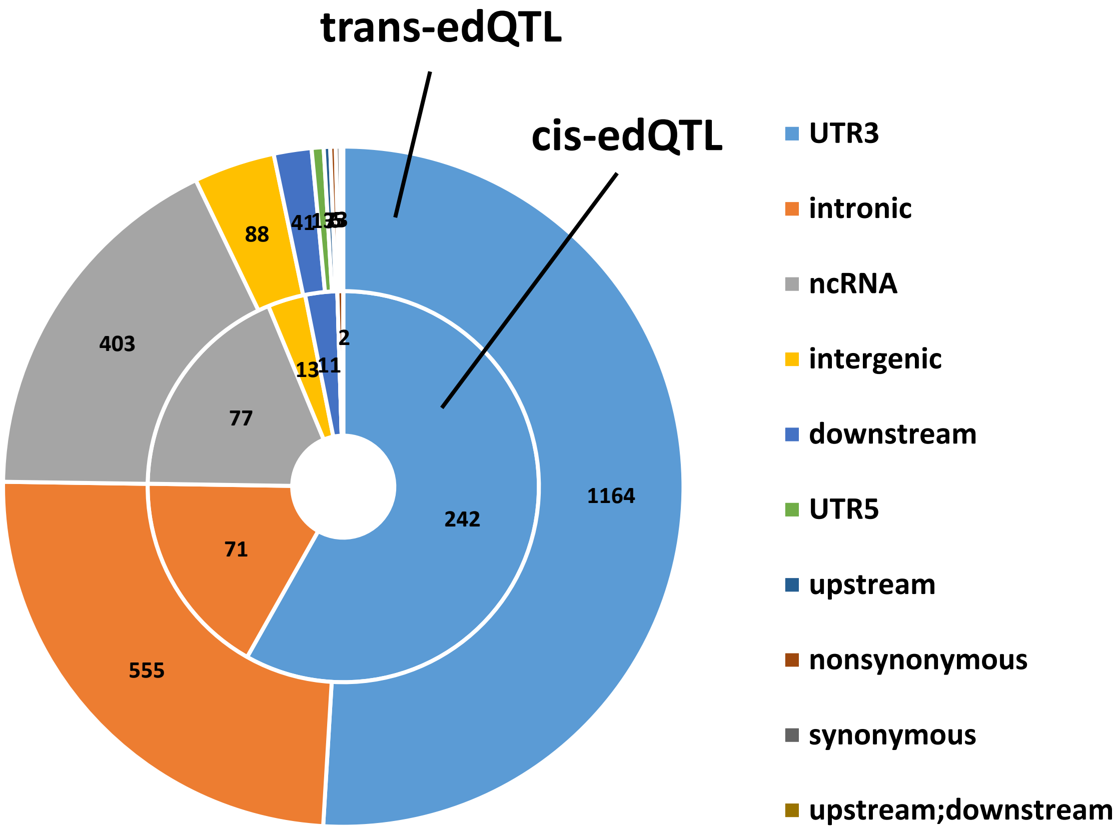
trans-edQTL
cis-edQTL
1164
555
403
88
41
242
71
77
13
11
2
UTR3
intronic
ncRNA
intergenic
downstream
UTR5
upstream
nonsynonymous
synonymous
upstream;downstream

Figure S6

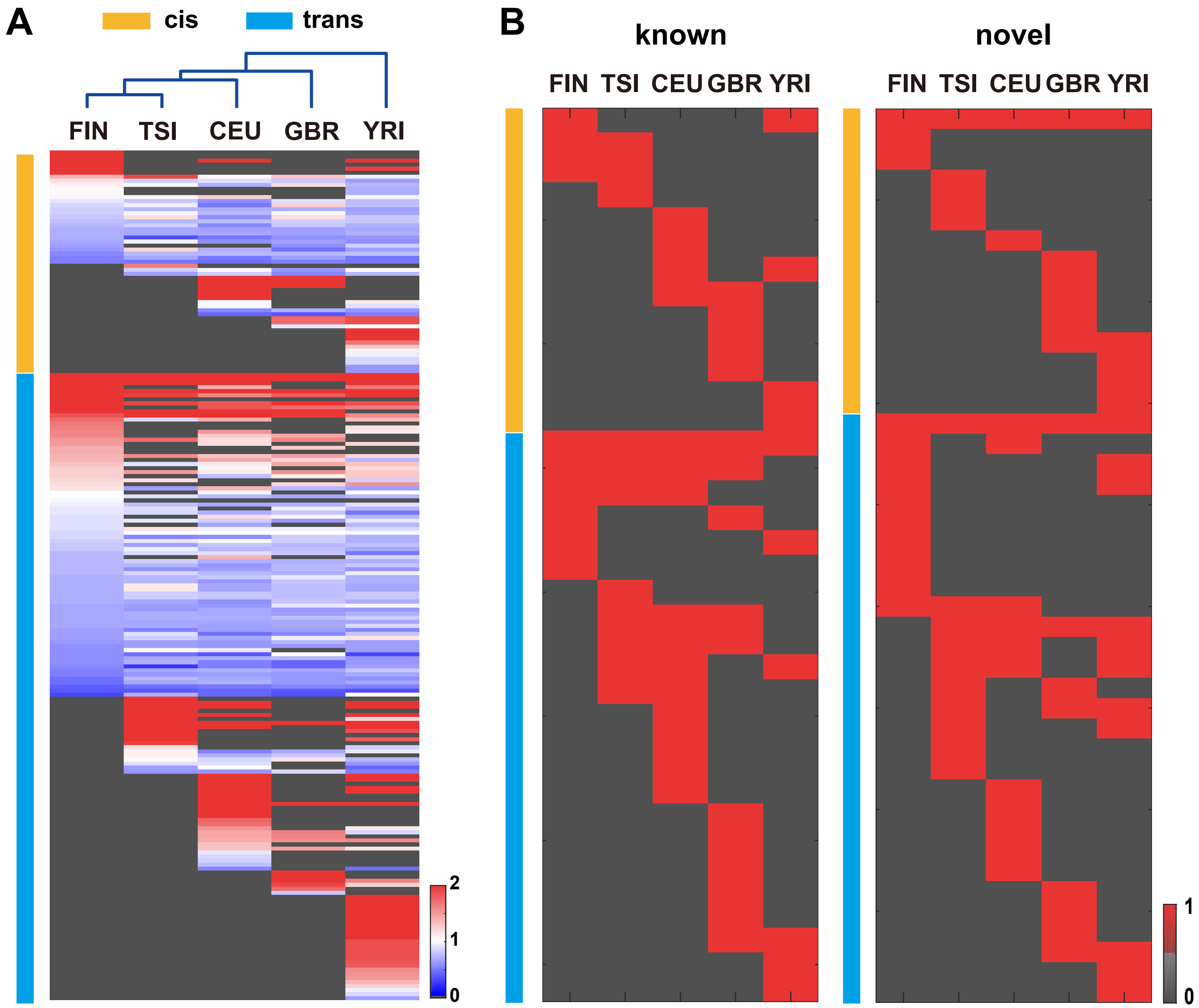
A
cis
trans
FIN
TSI
CEU
GBR
YRI
2
1
0
B
known
novel
FIN TSI CEU GBR YRI
FIN TSI CEU GBR YRI
1
0

Figure S7

A

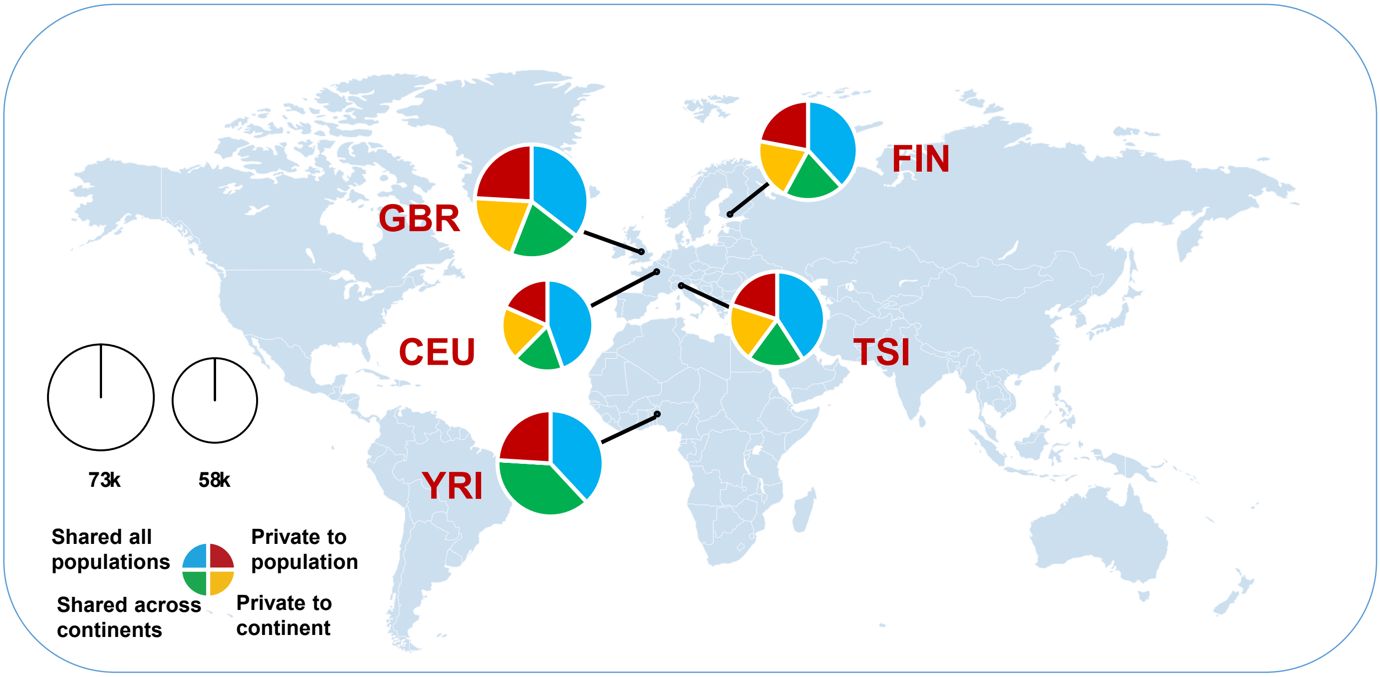
GBR
FIN
CEU
TSI
YRI
73k
58k
Shared all populations
Private to population
Shared across continents
Private to continent

B

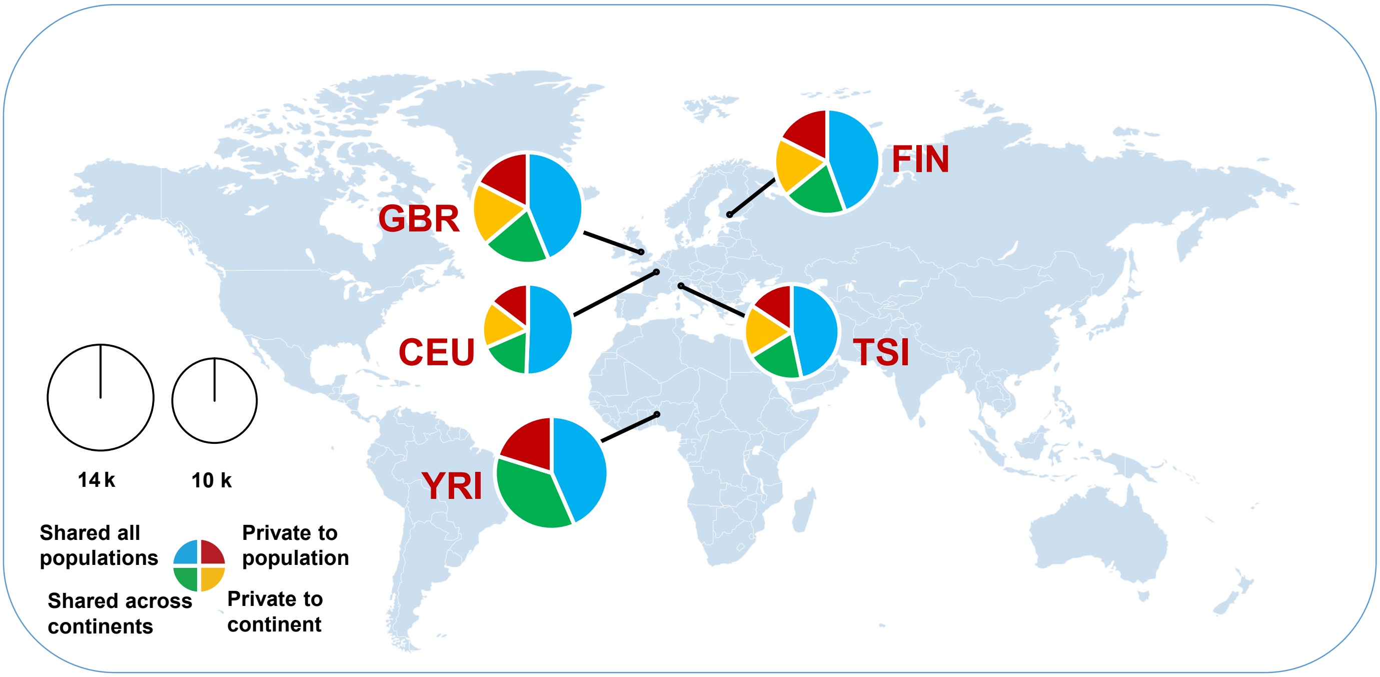
GBR
FIN
CEU
TSI
YRI
14 k
10 k
Shared all populations
Private to population
Shared across continents
Private to continent

Figure S8

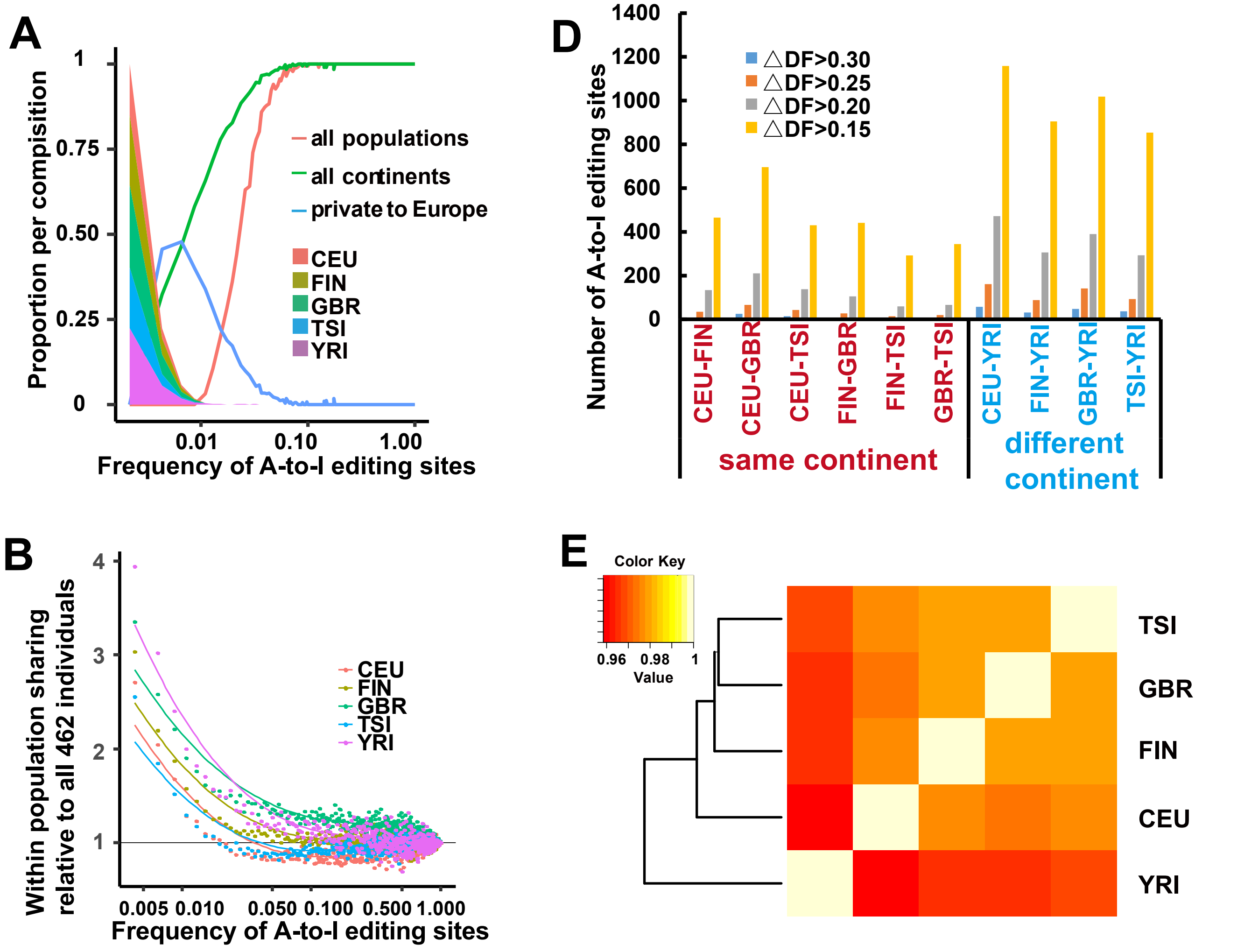

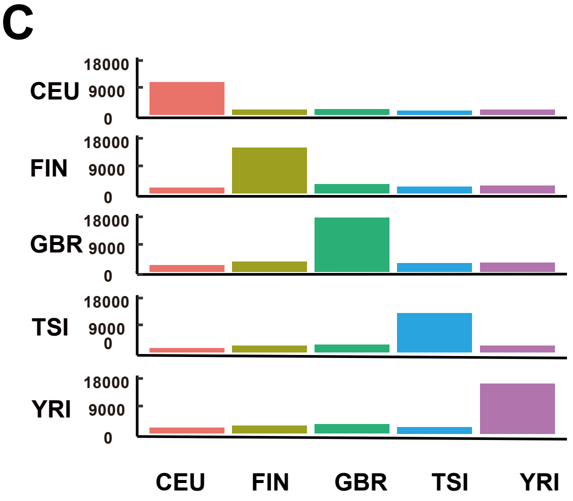

F

	YRI	CEU	FIN	GBR	TSI
YRI	0	5.14E-03	3.39E-03	3.91E-03	3.46E-03
CEU	5.14E-03	0	1.85E-03	2.76E-03	1.58E-03
FIN	3.39E-03	1.85E-03	0	1.18E-03	8.15E-04
GBR	3.91E-03	2.76E-03	1.18E-03	0	1.16E-03
TSI	3.46E-03	1.58E-03	8.15E-04	1.16E-03	0

Figure S9

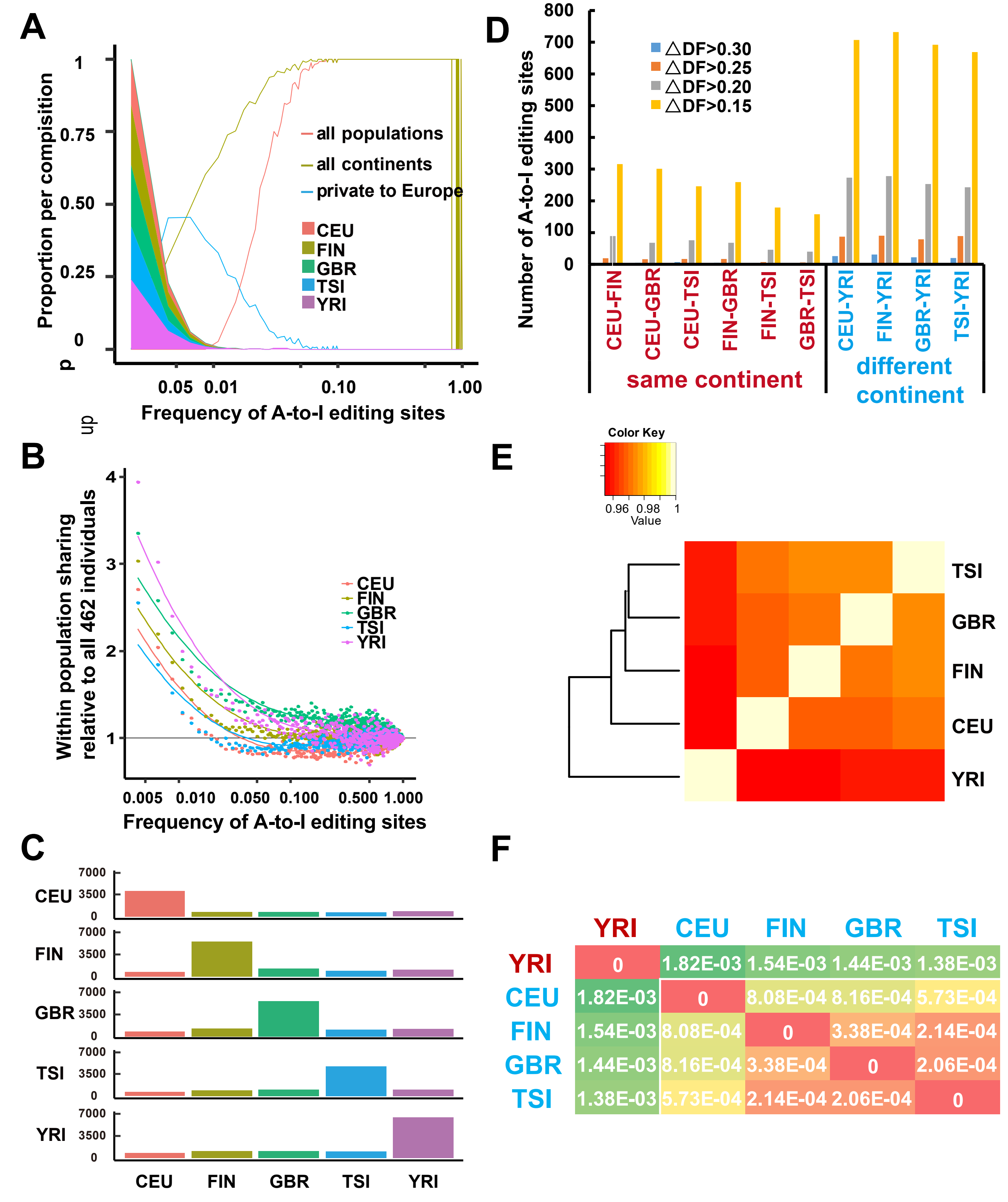

	YRI	CEU	FIN	GBR	TSI
YRI	0	1.82E-03	1.54E-03	1.44E-03	1.38E-03
CEU	1.82E-03	0	8.08E-04	8.16E-04	5.73E-04
FIN	1.54E-03	8.08E-04	0	3.38E-04	2.14E-04
GBR	1.44E-03	8.16E-04	3.38E-04	0	2.06E-04
TSI	1.38E-03	5.73E-04	2.14E-04	2.06E-04	0

Figure S10
